# Methyl Ether-Derivatized Sterols and Coprostanol Produced via Thermochemolysis Using Tetramethylammonium Hydroxide (TMAH)

**DOI:** 10.3390/molecules24224040

**Published:** 2019-11-07

**Authors:** Masatoshi Nakakuni, Yoshimi Yamasaki, Nonoka Yoshitake, Keiko Takehara, Shuichi Yamamoto

**Affiliations:** 1 Department of Science and Technology, Soka University, 1-236 Tangi-machi, Hachioji City, Tokyo 192-8577, Japan; 2 Department of Environmental Engineering for Symbiosis, Graduate School of Engineering, Soka University, 1-236 Tangicho, Hachioji, Tokyo 192-8577, Japan; e19m5711@soka-u.jp (Y.Y.); e19m5710@soka-u.jp (N.Y.); keiko-takehara@soka.gr.jp (K.T.); syama@soka.ac.jp (S.Y.)

**Keywords:** thermochemolysis, tetramethylammonium hydroxide (TMAH), sterols, coprostanol

## Abstract

Sterols are widely distributed in nature from lipids in organisms to sediments. As a conventional method, extraction and derivatization with TMS have been applied for sterol analysis, requiring a long preparation time for gas chromatography–mass spectrometry analysis. In this study, for sterol analysis, thermochemolysis using tetramethylammonium hydroxide (TMAH) was applied. This method performs hydrolysis and methylation simultaneously; thus, free and ether-bonding sterols can be analyzed as sterol methyl ethers in a relatively short time period. A sediment sample from a tideland (the Yatsu tideland, Japan) was analyzed using the TMAH method, and we detected more than 10 sterols, which include cholest-5-en-3*β*-ol (cholesterol), 24-ethylcholest-5-en-3*β*-ol (sitosterol), 24-methylcholesta-5,22*E*-3*β*-ol (brassicasterol), 24-ethylcholesta-5,24(28)*Z*-dien-3*β*-ol (isofucosterol), 4*α*,23,24-trimethyl-5*α*(H)-cholest-22*E*-en-3*β*- ol (dinosterol), and 5*β*(H)-cholestan-3*β*-ol (coprostanol). The detection of the various sterols can be attributed to multiple natural and artificial sources around the Yatsu tideland. In this paper, the mass spectra of these sterols are provided together with an interpretation of their fragmentation patterns. Additionally, the fecal pollution in the Yatsu tideland is discussed in the context of the detection of coprostanol.

## 1. Introduction

Sterols exist in the membranes of eukaryotic organisms, including microorganisms and terrestrial plants [1,2]. Thus, sterols have been detected in various natural environments, such as in lake and ocean waters and in sediments [3,4,5,6,7]. These natural sterols can be used to estimate ecological sources because sterol structures differ depending on the types of microorganisms [1,8]. Moreover, they have been used as environmental tracers, such as in degradation processes and for the reconstruction of paleoenvironments [9,10,11]. 

Sterols can be extracted with organic solvents, such as chloroform and methanol. A hydrolysis step is required if necessary. For sterol analysis by gas chromatography (GC)–mass spectrometry (MS), the extracted sterols must be derivatized to obtain enough volatility to pass through the GC. By derivatization, in the case of sterols, hydroxyl functional groups will be changed to more low-polarity functional groups. Typically, derivatization techniques are categorized into three types, namely, silylation, acylation, and alkylation. Although acylation is also used, silylation is most commonly used for derivatization.

**Figure 1:**
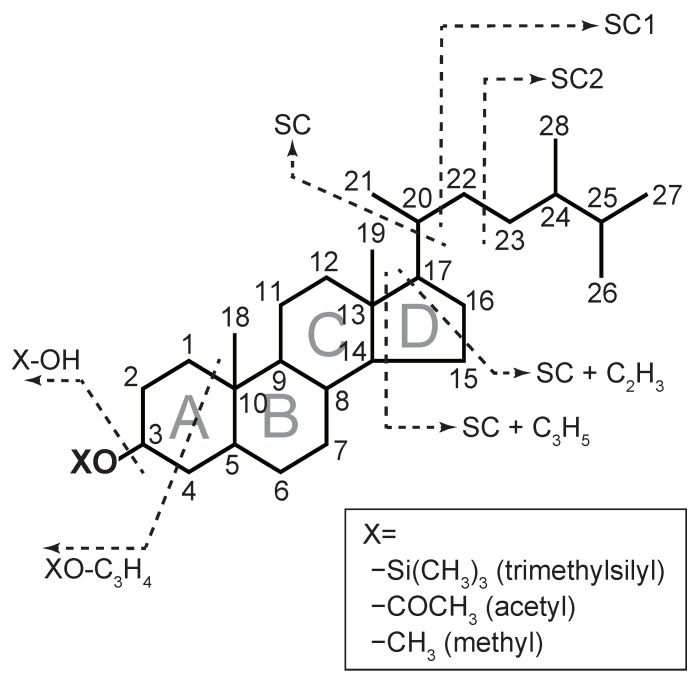
Diagnostic fragmentation of the sterols. Each number represents the position of the carbons. X varied with derivatization, for example, –Si(CH_3_)_3_ in trimethylsilyl, –COCH_3_ in acetyl, and –CH_3_ in methyl ethers. The X of the original sterols is H (i.e., –OH). SC refers to the side chain.

Silylation is effective for various functional groups, including carboxylic acids, amides, and alcohols, and it is performed using silylation reagents, such as *N*,*O*-bis(trimethylsilyl)trifluoroacetamide (BSTFA) and *N*,*O*-bis(trimethylsilyl)acetamide (BSA). The mass spectra of sterols determined via these conventional derivatizations have been reported in earlier studies [12,13,14,15,16]; thus, the interpretation of the mass spectra of sterols has become common, which allows us to easily identify sterols. Sterol trimethylsilyl (TMS) ether spectra can be divided into two patterns: ions that are accompanied by the loss of the side chain (SC) and ions that are not accompanied by the loss of the side chain (Figure 1). The loss of the side chain has five main variations: SC, SC1, SC2, SC + C_2_H_3_, and SC + C_3_H_5_. SC, SC1, and SC occur via the cleavage of C_17_–C_20_, C_20_–C_22_, and C_22_–C_23_ bonds, respectively. SC + C_2_H_3_ represents the cleavage of the side chain followed by the cleavage of C_13_–C_17_ and C_15_–C_16_ bonds (C_2_H_3_) on the D-ring. SC + C_3_H_5_ is from the cleavage of SC and C_13_–C_17_ and C_14_–C_15_ bonds (C_3_H_5_). However, the ions that are not accompanied by the loss of the SC only have two main variations: [HO–Si(CH_3_)_3_]^+^ and [(CH_3_)_3_Si–O–C_3_H_4_]^+^. Sterols can be identified by these fragmentations because these fragmentation patterns are different for each sterol. Meanwhile, silylation is very sensitive to water, causing degradation of the derivatized functional groups. Additionally, because Si in TMS reagents can remain in the inlet of the GC, there is a possibility that the inlet of the GC will be contaminated. Moreover, sample preparation before derivatization (e.g., the extraction step) requires a relatively long time.

Since it is a rapid method, thermochemolysis with tetramethylammonium hydroxide (TMAH) was applied for sterol analysis in this study. This method involves hydrolysis and methylation simultaneously, which allow the analysis of both ester and ether compounds and nonbonding compounds that have hydroxyl and carboxyl functional groups [17]. Thus, the TMAH method can provide various compounds, including lignin phenols, amino acids, sugars, and sterols, in one analysis in a relatively short time period [18,19,20,21,22,23]. Additionally, a recent study on the TMAH method extended the analysis to nucleobases [24]. Typically, thermochemolysis with TMAH includes using a pyrolyzer [25,26,27]. Several milligrams of the sample (the optimal weight is varied with the organic carbon content in the sample) was wrapped in a pyrofoil, and it was heated in a pyrolyzer that was directly connected to a GC–MS. Alternatively, thermochemolysis with TMAH can be carried out in a glass ampule [28]. Using this approach, thermochemolysis is performed on an ampule that contains a sample and the TMAH reagent that is sealed under vacuum in an oven.

**Figure 2:**
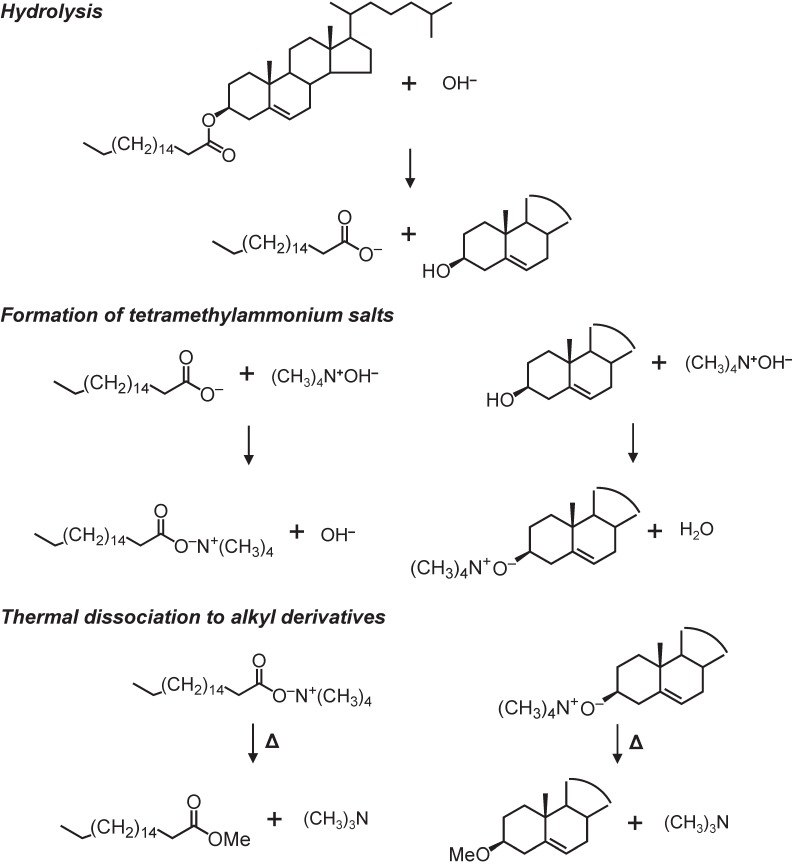
Generation processes for sterol methyl ether from cholesteryl stearate via the tetramethylammonium hydroxide (TMAH) reaction. This figure was drawn based on Asperger et al. [29] and Challinor [17].

Upon the TMAH reaction, sterols are provided as sterol methyl ethers. The application of the TMAH method for sterols can produce different types of sterols, including cholesterol, sitosterol, brassicasterol, and stanols [5,6,29]. The efficiency of methylation in sterols reaches >90% [29], indicating that sterols are able to be stably analyzed. Using the TMHA method, both free and bonding (e.g., cholesteryl ester) sterols can be analyzed (Figure 2). However, there are few reports on the interpretation of mass spectra of sterol methyl ethers, which could help detect them. Therefore, in this study, we provide the mass spectra of sterol ethers obtained using the TMAH method from a sediment sample and also provide an interpretation of these mass spectral patterns to show how to detect them.

## 2. Materials and Methods

### 2.1. A Tideland Surface Sediment Sample

**Figure 3:**
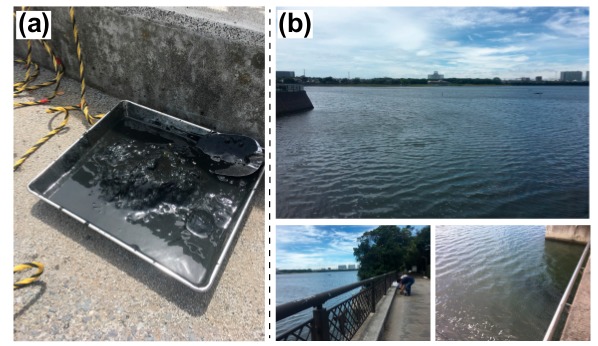
Pictures of the sampling point (Yatsu Tideland, Japan). (**a**) Picture of the sample. (**b**) State of the sampling place. The sample was taken from a bridge, as shown in the picture, using an Ekman–Birge grab.

In this study, the Yatsu tideland, Japan, was selected as the research location (Figure 3). The Yatsu tideland is located in the innermost location of Tokyo Bay, and is surrounded by industrial and residential areas that have expanded through reclamation works. In 1993, for the conservation of ecosystems and landscapes in the natural environment, the Yatsu tideland was registered in the Ramsar Convention. Various organisms, such as lugworms, gobies, clams, sea lettuces, and migratory birds inhabit the Yatsu Tideland [30,31,32,33], although the area is small (40 hectares). Therefore, various organic compound inputs are expected. A sediment sample was taken from the edge of the Yatsu tideland using an Ekman–Birge grab (Figure 3). The sediment sample was freeze-dried immediately after sampling and then powdered well. The powdered sample was stored in a freezer at −20 °C until the sterols were analyzed.

### 2.2. Thermochemolysis with TMAH

Several milligrams of the sample were added into a 20-mL glass ampule with 150 µL (25% in methanol) of TMAH reagent (Sigma–Aldrich Co., St. Louis, MO, USA) and with nonadecanoic-d37 acid (99.1%, CDN isotopes Co., Pointe-Claire, QC, Canada) as an internal standard. The glass ampule was placed into a desiccator to dry the solvents for approximately 15 min. To remove the solvents more sufficiently, a 40 °C hot plate with a nitrogen stream was applied for approximately 10 min. After the solvents were dried, the glass ampule was sealed under vacuum conditions and was heated in an oven at 300 °C for 30 min. After the ampule was cooled to room temperature, the reacted products were extracted with ethyl acetate thrice. The solvent was removed once, and the extracted products were re-resolved with ethyl acetate to an accurate volume of 100 µL. Several microliters within the re-resolved products were injected into the GC–MS system.

### 2.3. GC–MS Conditions

An Agilent GC–MS (GC, 6890N; MS, 5973 inert) was used for this study. A fused silica capillary GC column for the DB-5 ms (inner diameter, 0.25 mm; column length, 30 m; film thickness, 0.25 µm) was installed on the GC system with helium gas (purity: >99.9 vol.%) flowing as the carrier gas. The spitless injection mode was employed. The injection room temperature was set to 310 °C. The setting for the oven was as follows: 60 °C (20 min), 310 °C (6 °C/min), 310 °C (20 min). The mass ion source and quadrupole were programmed at 230 °C and 150 °C, respectively. MS data were recorded using the full scanning mode from 50 to 650 Da (Dalton). Ionization was achieved using the electron impact mode at 70 eV.

### 2.4. Identification of Sterols

The mass spectrum of the sterols obtained using the TMAH method was interpreted by comparing it with reported mass spectra from previous studies on methyl ether derivatization [34] and TMS derivatization [12,13,14,15]. The National Institute of Standards and Technology (NIST)/ United States Environmental Protection Agency (EPA)/ National Institutes of Health (NIH) Mass Spectral Library (NIST 05) was also employed for the interpretation of the sterols. The epicoprostanol-to-coprostanol and coprostanol-to-cholesterol ratios were calculated from their concentrations, which were obtained by comparison with the internal standard.

## 3. Results and Discussion

### 3.1. Identified Sterols in the Samples

**Figure 4:**
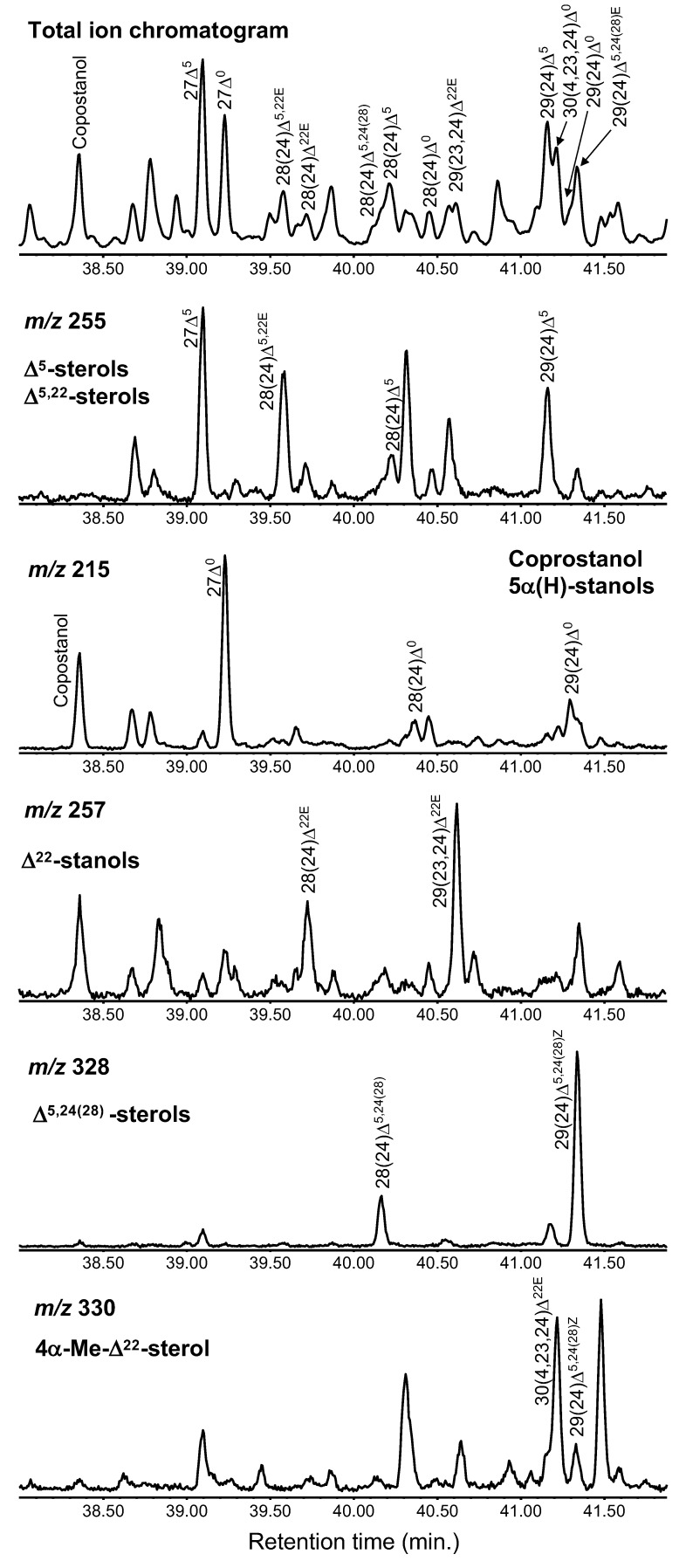
Partial total ion chromatogram and extracted ion chromatograms (*m*/*z* 255, 215, 257, 328, and 330) focused on sterols (38.00–41.88 min) via thermochemolysis using tetramethylammonium hydroxide (TMAH). The symbols are listed in Table 1.

From the Yatsu sediment, seven types of sterols from three Δ^5^-sterols, four 5*α*(H)-stanols, two 5*β*(H)-stanols, one Δ^5,22^-sterol, two Δ^22^-sterols, two Δ^5,24(28)^-sterols, and one 4*α*-Me-Δ^22^-sterol were determined (Table 1 and Figure 4). These multiple differences indicate that there are various input sources present for the Yatsu tideland, which can result from the rich ecosystem and human-living area being adjacent to each other.

### 3.2. Δ^5^-Sterols

**Figure 5:**
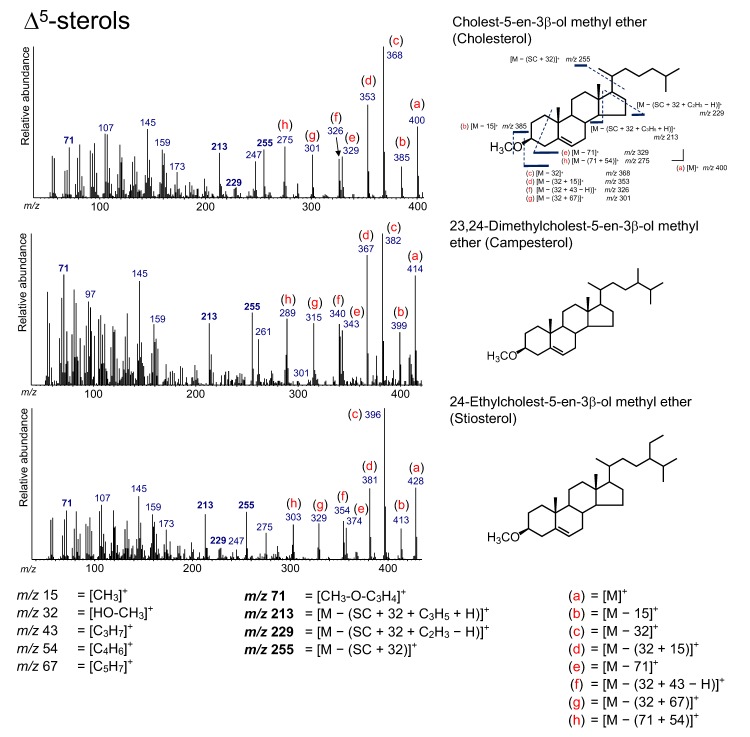
Electron ionization (70 eV) mass spectrum of Δ^5^-sterol. Bold numbers indicate an ion that accompanies the cleavage of the side chain. (**a**)–(**h**) Ions that are not accompanied by the loss of the side chain or other groups.

The compounds of cholest-5-en-3*β*-ol (cholesterol), 24-methyl-cholest-5-en-3*β*-ol (campesterol), and 24-ethylcholest-5-en-3*β*-ol (sitosterol) were detected from the tidal sediment, and they were grouped as Δ^5^-sterols (Figure 4 and Figure 5). C_27_ and C_28_ sterols, including cholesterol and campesterol, are widely present in plankton [1,8]. However, C_29_ sterols, such as sitosterol, are present in terrestrial plants [35,36]. Therefore, the distributions of C_27_, C_28_, and C_29_ sterols have been used for the estimation of sources in sedimentary sterols [8]. However, the results must be interpreted carefully, because C_29_ sterols are also found in phytoplankton [1].

A peak at *m*/*z* 71, a characteristic ion for Δ^5^-sterol, was observed for all Δ^5^-sterols, representing [CH_3_–O–C_3_H_4_]^+^. Peaks at *m*/*z* 255, 229, and 213 were due to an ion accompanying the loss of the SC (Figure 5). These peaks (*m*/*z* 71, 255, 229, and 213) did not change with the type of derivatization because these peaks appear together with the loss of a derivatized functional group at the C_3_ position. Therefore, these peaks have also been found in Δ^5^-sterol TMS derivatives [12,13,14]. The peak at *m*/*z* 255 is derived from the losses of the side chain and HO–CH_3_ from the Δ^5^-sterols ([M − (SC + 32)]^+^). In addition, the peak at *m*/*z* 229 results from the loss of C_2_H_3_ (without a hydrogen atom) from the D-ring ([M − (SC + 32 + C_2_H_3_ − H)]^+^). The peak at *m*/*z* 213 is from the loss of *m*/*z* 255 from the molecular ion of Δ^5^-sterols followed by the loss of C_3_H_5_ (with a hydrogen atom) on the D-ring ([M − (SC + 32 + C_3_H_5_ + H)]^+^). The peaks at *m*/*z* 255 and *m*/*z* 213 are clear in all the Δ^5^-sterols detected, although the peak at *m*/*z* 229 is weak for campesterol.

[M]^+^, [M − 15]^+^, [M − 32]^+^, [M − (32 + 15)]^+^, [M − 71]^+^, [M − (32 + 67)]^+^, [M − (71 + 54)]^+^, and [M − (71 + 82)]^+^ are all ions that were not accompanied by the loss of the SC (Figure 5). These ions were also confirmed from the fragmentation of sterol TMS derivatives, although their intensities in methyl ethers are relatively high than in TMS ethers. [M − 15]^+^ is produced by the loss of a methyl group from sterols. [M − 32]^+^ represents the loss of HO–CH_3_. The additional loss of a methyl group and C_5_H_7_ from [M − 32]^+^ yields [M − (32 + 15)]^+^ and [M − (32 + 67)]^+^, respectively. [M – 71]^+^ arises from the loss of CH_3_–O–C_3_H_4_. Additionally, [M − (71 + 54)]^+^ and [M − (71 + 82)]^+^ are generated by an additional loss of C_4_H_6_ on the A-ring or C_6_H_10_ on the A- and B-rings, respectively. [M − (32 + 43 − H)]^+^ can result from the loss of HO–CH_3_ from Δ^5^-sterols, followed by the loss of C_3_H_7_ (without a hydrogen atom) at the end of the SC (Figure 5).

### 3.3. 5α(H)-Stanols

**Figure 6:**
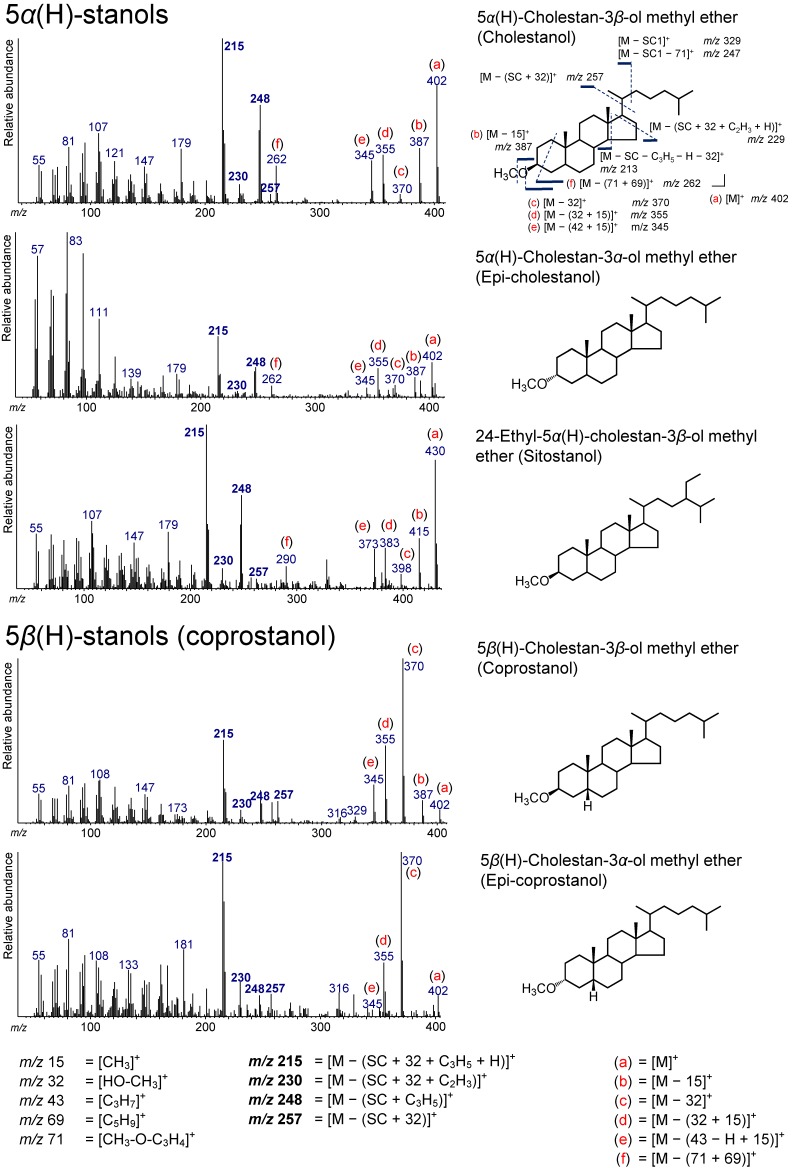
Electron ionization mass spectra of 5*α*(H)-stanol and 5*β*(H)-stanol (coprostanol). Bold numbers indicate an ion that accompanies the cleavage of the side chain. (**a**)–(**f**) Ions that not accompanied by the loss of the side chain or other groups.

Four 5*α*(H)-stanols, 5*α*(H)-cholestan-3*α*-ol (epi-cholestanol), 5*α*(H)-cholestan-3*β*-ol (cholestanol), 24-methyl-5*α*(H)-cholestan-3*β*-ol (campestanol), and 24-ethyl-5*α*(H)-cholestan-3*β*-ol (sitostanol) were determined (Figure 4 and Figure 6). Typically, 5*α*(H)-stanols are produced under anoxic conditions via bacterial conversion from sterols [7,37,38]. Thus, the ratio of 5*α*(H)-stanol/Δ^5^-sterol can be applied as a tracer for redox conditions in the field of organic geochemistry [5,6,39]. However, the 5*α*(H)-stanol/Δ^5^-sterol ratio could be large due to the preferential degradation of sterols in terrestrial organic matters, even under oxic conditions [35].

The peaks at *m*/*z* 257, 248, 230, and 215 for 5*α*(H)-stanols are due to an ion accompanying the loss of the SC in 5*α*(H)-stanols (Figure 6). The peak at *m*/*z* 248 is derived from the losses from the side chain and the D-ring from 5*α*(H)-stanols ([M – (SC + C_3_H_5_)]^+^). The ion at *m*/*z* 257 represents the losses of HO–CH_3_ and the side chain ([M – (SC + 32)]^+^), and the ion at *m*/*z* 230 is produced by the additional loss of the D-ring from the ion at *m*/*z* 257 ([M – (SC + 32 + C_2_H_3_)]^+^). The peak at *m*/*z* 215 is a base peak in 5*α*(H)-stanols and is due to the further loss of the D-ring (with a hydrogen atom) from the *m*/*z* 257 ion ([M – (SC + 32 + C_3_H_5_ + H)]^+^). As an ion that is not accompanied by the loss of the SC, for example, in cholestanol, peaks at *m*/*z* 402 ([M]^+^), 387 ([M − 15]^+^), 370 ([M − 32]^+^), and 355 ([M − (32 + 15)]^+^) were confirmed (Figure 6). The peaks appearing at *m*/*z* 345 and 262 in the spectrum of cholestanol were not found in the spectra of the TMS ethers (Figure 6). The peak at *m*/*z* 345 may originate from the loss of C_3_H_7_ from the SC and a methyl group ([M – (42 + 15)]^+^). Additionally, the peak at *m*/*z* 262 can be from the loss of CH_3_–O–C_3_H_4_ followed by the loss of C_5_H_9_ at the A- and B-rings ([M − (71 + 69)]^+^). Although campestanol was also confirmed, the spectrum is not shown in Figure 6, since it was ambiguous.

### 3.4. 5β(H)-Stanols (Coprostanol and Epicoprostanol)

From the sediment sample, 5*β*(H)-cholestan-3*β*-ol (coprostanol) and 5*β*(H)-cholestan-3*α*-ol (epicoprostanol) were determined (Figure 4 and Figure 6). Coprostanol can be produced through the reduction of cholesterol by bacteria within the gut of higher animals including humans [40,41], which is ubiquitously present in their fecal matter [42,43]. Therefore, coprostanol has been used as an indicator of fecal pollution and has been widely applied to environments [30,44,45,46,47,48,49]. 

The mass fragments that appeared for coprostanol and epicoprostanol were similar to those of the 5*α*(H)-stanols, implying that the same fragmentation as in 5*α*(H)-stanols occurs in 5*β*(H)-stanols (Figure 6). However, these intensities are considerably different from those of 5*α*(H)-stanols. As with 5*α*(H)-stanols, the peaks at *m*/*z* 257, 248, 230, and 215 were confirmed from coprostanol and epicoprostanol as an ion accompanying the cleavage of the SC (Figure 6). An intense peak at *m*/*z* 248 represents C_3_H_5_ in the D-ring according to the molecular weight followed by the loss of the side chain ([M – (SC + C_3_H_5_)]^+^). The peak at *m*/*z* 257 is derived from the loss of HO–CH_3_ and the side chain ([M – (32 + SC)]^+^), and the peak at *m*/*z* 230 is from an additional loss of C_2_H_3_ in the D-ring from the *m*/*z* 257 ion ([M – (SC + 32 + C_2_H_3_)]^+^). The peak at *m*/*z* 215 represents the loss of C_3_H_5_ (with molecular hydrogen) in the D-ring from the *m*/*z* 257 ion ([M − (SC + 32 + C_3_H_5_ + H)]^+^), which is a characteristic ion for 5*α*(H)- and 5*β*(H)-stanols [50,51,52]. Additionally, in the spectrum of coprostanol and epicoprostanol, the peaks at *m*/*z* 402 ([M]^+^), 387 ([M − 15]^+^), 370 ([M − 32]^+^), and 355 ([M − (32 + 15)]^+^) were confirmed to be due to ions that are not accompanied by the loss of the SC (Figure 6).

**Figure 7:**
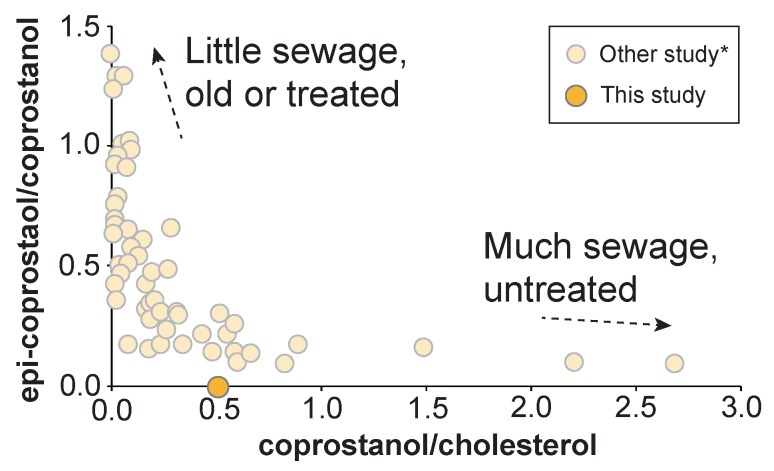
A biplot of the coprostanol/cholesterol ratio with the epicoprostanol/coprostanol ratio for the Yatsu sediment. Our data is plotted on a redrawn figure from Mudge and Seguel [54] and Mudge and Ball [55].

The detection of coprostanol from sediment suggests that the Yatsu tideland is affected by some fecal pollution. This might be because industrial and residential areas were expanded by reclamation works, and they have enhanced the environmental load on the Yatsu tideland. A biplot of the coprostanol/cholesterol ratio with the epicoprostanol/coprostanol ratio in the sediment is shown in Figure 7. Epicoprostanol is typically converted from coprostanol via a bacterial reaction during sewage treatment processes, and therefore is observed from treated or old sewage samples [53]. Thus, a high epicoprostanol/coprostanol ratio is observed from “old or treated” sewage samples [54]. In our Yatsu tideland sediment sample, the epicoprostanol/coprostanol ratio shows approximately 0.4, indicating that the source is a mixture of new and old. The ratio of coprostanol/cholesterol can estimate the degree of fecal contamination [55,56,57]. According to a proposal by Grimalt and Albaigés [56], when the coprostanol/cholesterol ratio is greater than 0.2 in an environmental sample, the environment can be considered to have fecal contamination. The coprostanol/cholesterol ratio in our sediment sample was approximately 0.5 (Figure 7), which is over the value of the definition. Therefore, the Yatsu tideland is an environment that is affected by fecal pollution according to this definition. However, the value is not higher as with large contaminated areas (Figure 7). Additionally, our data is limited to one site. Thus, a more accurate investigation of the polluted area of the Yatsu tideland requires that different sample areas be analyzed.

Of note, coprostanol analysis via the TMAH method has methodological advantages because the process can be performed quickly with a small amount of solvent. These advantages can result in the treatment of a large number of samples and the reduction of solvents during analysis.

### 3.5. Δ^5,22^-Sterol (Brassicasterol)

**Figure 8:**
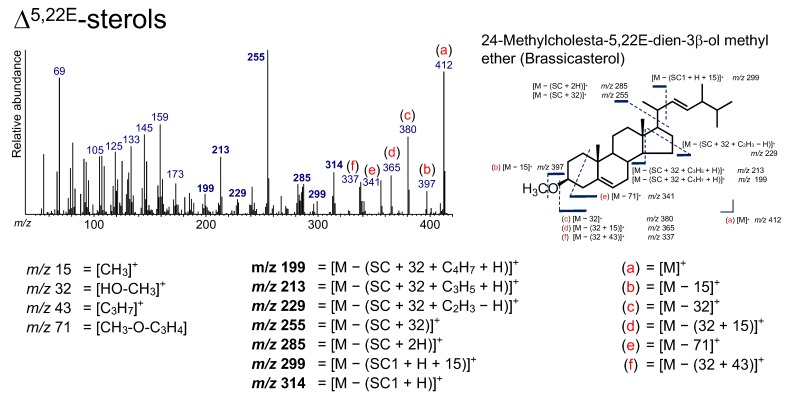
Electron ionization mass spectrum of 24-methylcholesta-5,22*E*-dien-3*β*-ol methyl ether (brassicasterol). Bold numbers indicate an ion accompanying the cleavage of the side chain. (**a**)–(**f**) Ions that are not accompanied by the loss of the side chain or other groups.

A Δ^5,22^-sterol, 24-methylcholesta-5,22*E*-dien-3*β*-ol (brassicasterol), was determined from the sediment (Figure 4 and Figure 8). Brassicasterol is present in diatoms as a major sterol [58,59,60]. In particular, pennate diatoms have a high relative abundance of brassicasterol (>60% of sterols) [61]. However, this sterol cannot be a specific biomarker for diatoms because the sterol is present in other algal groups [61].

The peaks at *m*/*z* 314 ([M − (SC1 + H)]^+^), 299 ([M − (SC1 + H + 15)]^+^), 285 ([M − (SC + 2H)]^+^), 255 ([M − (SC + 32)]^+^), 229 ([M − (SC + 32 + C_2_H_3_ − H)]^+^), 213 ([M − (SC + 32 + C_3_H_5_ + H)]^+,^), and 199 ([M − (SC + 32 + C_4_H_7_ + H)]^+^) in the brassicasterol are due to ions that accompany the cleavage of the SC (Figure 8). These ions are present in the spectra of Δ^5^-sterols and Δ^22^-sterols as well. Additionally, the peaks at *m*/*z* 255 and 213 were also found in the TMS derivative, because these ions do not include a derivatized functional group [12,62]. However, peaks at *m*/*z* 412 ([M]^+^), 397 ([M − 15]^+^), 380 ([M − 32]^+^), 365 ([M − (32 + 15)]^+^), 341 ([M − 71]^+^), and 337 ([M − (32 + 43)]^+^) were found to result not by the loss of the SC, and they are also observed for Δ^5^-sterols and Δ^22^-sterols (Figure 8). The peak at *m*/*z* 125 represents the molecular weight of its SC. These ions are important for the determination of Δ^5,22^-sterols.

### 3.6. Δ^22^-Stanols

**Figure 9:**
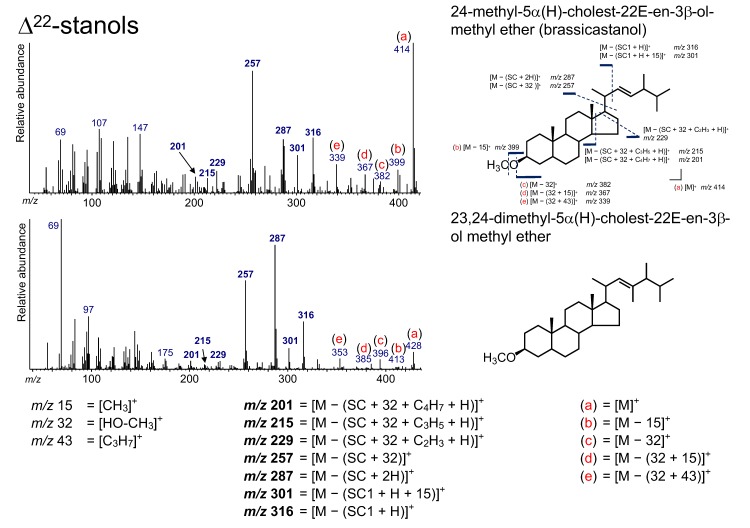
Electron ionization mass spectrum of Δ^22^-stanol. Bold numbers indicate an ion that accompanies the cleavage of the side chain. (**a**)–(**e**) Ions that are not accompanied by the loss of the side chain or other groups.

Two Δ^22^-stanols, 24-methyl-5*α*(H)-cholest-22*E*-en-3*β*-ol (brassicastanol) and 23,24-dimethyl-5*α*(H)-cholest-22*E*-en-3*β*-ol, were determined from the sediment (Figure 4 and Figure 9). As with 5*α*(H)-sterols, Δ^22^-stanols can be produced from the bacterial conversion from Δ^5,22^-sterols. Indeed, a sterol analysis of particle matter at the oxic–anoxic boundary in the Black Sea implied that the brassicastanol/brassicasterol ratio increases at the anoxic water layer [7]. Additionally, a continuous record of the brassicastanol/brassicasterol ratio from California late Quaternary sediments has shown historical changes in redox conditions over 40 kyr [5].

The peaks at *m*/*z* 316, 301, 287, 257, 229, 215, and 201 are due to ions that accompany the cleavage of the SC (Figure 9). The peak at *m*/*z* 316 results from the loss of the side chain (SC1) with a hydrogen atom from Δ^22^-stanols ([M – (SC1 + H)]^+^), and the additional loss of a methyl group yields the ion at *m*/*z* 301 ([M – (SC1 + H + 15]^+^). The peak at *m*/*z* 287 originates from the loss of the side chain (with two hydrogen atoms) from Δ^22^-stanols ([M – (SC + 2H)]^+^). An intense peak at *m*/*z* 257 is derived from the loss of HO–CH_3_ and the side chain from Δ^22^-stanols ([M – (32 + SC)]^+^). This ion, *m*/*z* 257, is a characteristic ion that is used for the determination of Δ^22^-sterols, as in the case of TMS derivatization [51,63,64], and it shows that the sterol skeleton is saturated (no double bond on the C_5_–C_6_ position) as with 5*α*(H)-stanols [51]. The peaks at *m*/*z* 229, 215, and 201 can arise from the loss of C_2_H_3_ (with a hydrogen atom) at the D-ring ([M – (32 + SC + C_2_H_3_ + H)]^+^), the loss of C_3_H_5_ (with a hydrogen atom) at the D-ring ([M – (32 + SC + C_3_H_5_ + H)]^+^), and C_4_H_7_ (with a hydrogen atom) at the D- and C-rings from the ion at *m*/*z* 257 ([M – (32 + SC + C_4_H_7_ + H)]^+^). Ions [M]^+^, [M − 15]^+^, [M − 32]^+^, [M − (32 + 15)]^+^, and [M − (32 + 43)]^+^ were confirmed to be not accompanied by the loss of the SC (Figure 9). [M − (32 + 43)]^+^ can arise from the loss of HO–CH_3_ from Δ^22^-stanols, which is followed by the loss of C_3_H_7_ on the end of the SC.

### 3.7. Δ^5,24(28)^-Sterols

Two Δ^5,24(28)^-sterols, 24-methylcholesta-5,24(28)-dien-3*β*-ol (24-methylenecholesterol) and 24-ethyl-cholesta-5,24(28)*Z*-dien-3*β*-ol (isofucosterol), were determined from the sediment (Figure 3 and Figure 9). 24-methylenecholesterol is present in some microalgae, such as diatoms [1], which are found in various aquatic environments including in the Arctic and Antarctic oceans [65,66,67]. Isofucosterol has been observed in green microalgae as a major sterol [1].

**Figure 10:**
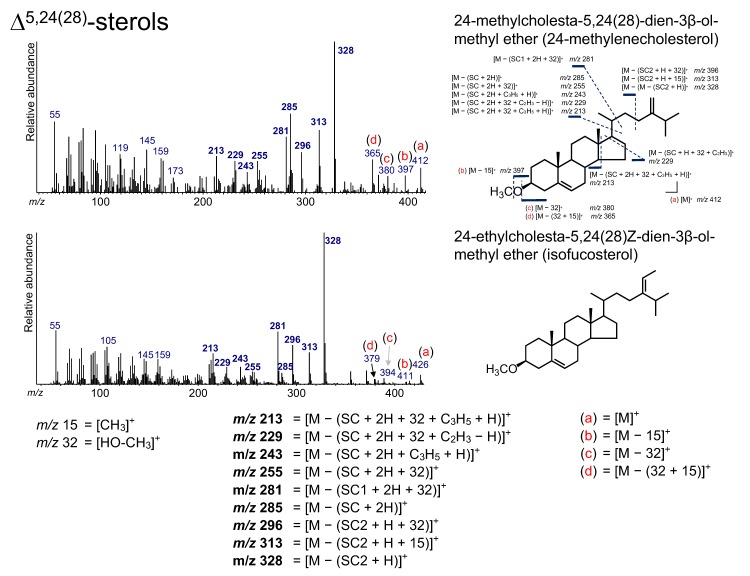
Electron ionization mass spectrum of Δ^5,24(28)^-sterol. Bold numbers indicate an ion that accompanies the cleavage of the side chain. (**a**)–(**d**) Ions that are not accompanied by the loss of the side chain.

The spectra of both Δ^5,24(28)^-sterols show peaks at *m*/*z* 328, 313, 296, 285, 281, 255, 243, 229, and 213 (Figure 10). Of these ions, the peaks at *m*/*z* 328, 313, 285, and 243 were also confirmed in the spectra of the Δ^24(28)^-sterols. The ion at *m*/*z* 328 arises from the loss of the side chain (SC2) with a hydrogen atom and is also a characteristic ion for Δ^5,24(28)^-sterols. The peaks at *m*/*z* 255, 253, 229, and 213 are also visible in the fragmentation pattern of other sterols, such as Δ^24(28)^-sterols and Δ^5^-sterols. The peak at *m*/*z* 296 results from the losses of the side chain (SC1) and HO–CH_3_ from Δ^5,24(28)^-sterols ([M − (SC1 + 32)]^+^). The additional elimination of a methyl group yields *m*/*z* 281 ([M − (SC1 + 32 + 15)]^+^). Ions [M]^+^, [M − 15]^+^, [M − 32]^+^, and [M − (32 + 15)]^+^ were confirmed to be ions that are not accompanied by the loss of the SC, which are also present for other sterols, including Δ^5^-sterols (Figure 10).

### 3.8. 4α-Me-Δ^22^ Sterol

**Figure 11:**
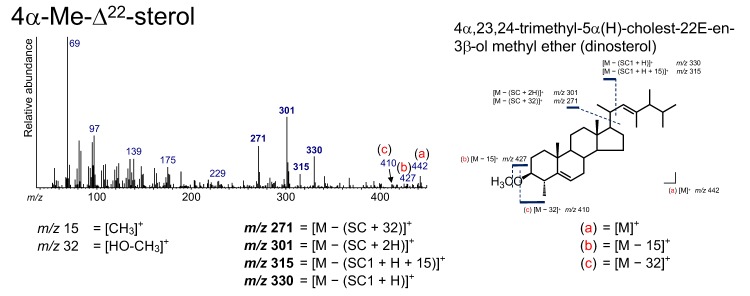
Electron ionization mass spectrum of 4*α*,23,24-trimethyl-5*α*(H)- cholest-22*E*-en-3*β*-ol methyl ether (dinosterol). Bold numbers indicate an ion that accompanies the cleavage of the side chain. (**a**)–(**c**) Ions that are not accompanied by the loss of the side chain.

A 4*α*-Me-Δ^22^ sterol, 4*α*,23,24-trimethyl-5*α*(H)-cholest-22*E*-en-3*β*-ol (dinosterol), was identified from the sediment (Figure 4 and Figure 11). Sterols having a methyl functional group at the C_4_ position, including dinosterol, are abundant in dinoflagellates [68,69,70]. Therefore, dinosterol has been used as a unique biomarker for dinoflagellates [71,72].

In the spectrum of dinosterol, [M]^+^, [M − 15]^+^, [M − 32]^+^, *m*/*z* 330, 315, 301, 271, 245, and 229 were confirmed, which can be interpreted by the 14 Da (CH_3_) higher shift of the corresponding fragmentation patterns in the spectrum of 23,24-dimethyl-5*α*(H)-cholest-22*E*-en-3*β*-ol methyl ether (Figure 11).

### 3.9. Identification of Sterols from the Extracted Ion Chromatograms

Determining each sterol from the GC–MS results can be effectively achieved from the extracted ion chromatograms of each sterol. The ion at *m*/*z* 255 is useful to determine Δ^5^-, Δ^5,22^-, and Δ^5,24(28)^-sterols. Ion chromatograms with *m*/*z* 215, 257, 328, and 330 represent 5*α*- and 5*β*-stanols, Δ^22^-stanols, Δ^5,24(28)^-sterols, and 4*α*-Me-Δ^22^-sterols, respectively (Figure 4).

The retention time in the GC–MS results is also important for determining sterols. For example, coprostanol and cholestanol, isomers of the H atom at the C_5_ position (i.e., 5*β*(H) or 5*α*(H)), elute at substantially different times even though the structures are similar (Figure 4). The retention time of coprostanol is earlier than that of cholestanol. Additionally, 5*α*(H)-stanols and Δ^22^-stanols were obtained immediately after their corresponding Δ^5^-sterols and Δ^5,22^-stanols, which is useful for the determination of these compounds (Figure 4). Additionally, the number of double bonds and their positions (C_22_ and/or C_5_ positions) appear at different retention times; for example, retention times become longer in the order of 28(24)Δ^5,22*E*^ (brassicasterol), 28(24)Δ^22*E*^ (brassicastanol), and 28(24)Δ^5^ (campesterol) (Figure 4).

## 4. Conclusions

A tideland surface sediment from the Yatsu tideland, Japan, was analyzed using thermochemolysis with tetramethylammonium hydroxide (TMAH) for sterols. From the sediment, 10 sterols, including Δ^5^-sterols, 5*α*(H)-sterols, Δ^5,22^-sterols, Δ^22^-sterols, Δ^5,24(28)^-sterols, 4*α*-Me-Δ^22^-sterols, and coprostanol, were determined. The Yatsu tideland sediment sample enabled the determination of these various sterols, because the Yatsu tideland is located where rich ecosystems and human-living areas are adjacent to each other. The MS results of these sterols helped determine them. Characteristic mass fragments at *m*/*z* 255, *m*/*z* 215, *m*/*z* 257, and *m*/*z* 330 were useful to detect Δ^5^-type sterols (i.e., Δ^5^-sterols, Δ^5,22^-sterols, and Δ^5,24(28)^-sterols), 5*α*(H)-sterols, 5*β*(H)-sterols, Δ^22^-sterols, and 4*α*-Me-Δ^22^-sterols from the extracted mass chromatograms. Additionally, some fecal pollution in the Yatsu tideland was suggested by the detection of coprostanol. The TMAH method can treat samples in a relatively short time with a small amount of sample and then provide sterol methyl ethers. Thus, this method is suitable for studies that analyze many samples. The provided sterol spectra in this study, including other such studies, will help determine the kinds of sterols present in a sample using the GC–MS result.

## Figures and Tables

**Table 1:** Sterols identified from the Yatsu surface sediment.

Retention Time (min)	Symbol *	Systematic Name	Trivial Name	Formula (Methylated Ether)	Molecular Weight (Methylated Ether)	*m*/*z* **
38.358	Coprostanol	5*β*(H)-Cholestan-3*β*-ol methyl ether	Coprostanol	C_28_H_50_O	402	215, 230, 248, 257, 345, 355, **370**, 387, *402*
38.671	Epicoprostanol	5*β*(H)-Cholestan-3*α*-ol methyl ether	Epicoprostanol	C_28_H_50_O	402	215, 230, 248, 257, 345, 355, **370**, 387, *402*
38.777	Epicholestanol	5*α*(H)-Cholestan-3*α*-ol methyl ether	Epicholestanol	C_28_H_50_O	402	**215**, 230, 248, 257, 345, 355, 370, 387, *402*
39.096	27Δ^5^	Cholest-5-en-3*β*-ol methyl ether	Cholesterol	C_28_H_48_O	400	213, 229, 255, 275, 301, 326, 329, 353, **368**, 385, *400*
39.228	27Δ^0^	5*α*(H)-Cholestan-3*β*-ol methyl ether	Cholestanol	C_28_H_50_O	402	**215**, 230, 248, 257, 345, 355, 370, 387, *402*
39.578	28(24)Δ^5,22*E*^	24-Methylcholesta-5,22*E*-dien-3*β*-ol methyl ether	Brassicasterol	C_29_H_48_O	412	199, 213, 229, **255**, 285, 299, 314, 337, 341, 365, 380, 397, *412*
39.722	28(24)Δ^22*E*^	24-Methyl-5*α*(H)-cholest-22*E*-en-3*β*-ol methyl ether	Brassicastanol	C_29_H_50_O	414	201, 215, 229, **257**, 287, 301, 316, 339, 367, 382, 399, *414*
40.166	28(24)Δ^5,24(28)^	24-Methylcholesta-5,24(28)-dien-3*β*-ol methyl ether	24-Methylenecholesterol	C_29_H_48_O	412	213, 229, 243, 255, 281, 285, 296, 313, **328**, 365, 380, 397, *412*
40.229	28(24)Δ^5^	24-Methylcholest-5-en-3*β*-ol methyl ether	Campesterol	C_29_H_50_O	414	213, 255, 261, 289, 301, 315, 340, 343, 367, **382**, 399, *414*
40.360	28(24)Δ^0^	24-Methyl-5*α*(H)-cholestan-3*β*-ol methyl ether	Campestanol	C_29_H_52_O	416	**215**, 230, 248, 257, 359, 369, 384, 401, *416*
40.616	29(23,24)Δ^22*E*^	23,24-Dimethyl-5*α*(H)-cholest-22*E*-en-3*β*-ol methyl ether		C_30_H_52_O	428	201, 215, 229, **257**, 287, 301, 316, 353, 385, 396, 413, *428*
41.161	29(24)Δ^5^	24-Ethylcholest-5-en-3*β*-ol methyl ether	Sitosterol	C_30_H_52_O	428	213, 229, 255, 275, 303, 329, 354, 357, 381, **396**, 413, *428*
41.217	30(4,23,24)Δ^22*E*^	4*α*,23,24-Trimethyl-5*α*(H)-cholest-22*E*-en-3*β*-ol methyl ether	Dinosterol	C_31_H_54_O	442	271, **301**, 315, 330, 410, 427, *442*
41.298	29(24)Δ^0^	24-Ethyl-5*α*(H)-cholestan-3*β*-ol methyl ether	Sitostanol	C_30_H_54_O	430	**215**, 230, 248, 257, 290, 373, 383, 398, 415, *430*
41.336	29(24)Δ^5,24(28)*Z*^	24-Ethylcholesta-5,24(28)*Z*-dien-3*β*-ol methyl ether	Isofucosterol	C_30_H_50_O	426	213, 229, 243, 255, 281, 285, 296, 313, **328**, 379, 394, 411, *426*

* The symbols are given as n(m)Δ^p^, where n is the carbon number of a sterol, m is the position of the methyl or ethyl group in the side chain, and p is the position of a double bond (s). ** *m*/*z*: bold and italic denote the base peak and molecular ion, respectively.

## Founding

The research was financially supported by the Sasakawa Scientific Research Grant (2018) from The Japan Science Society.
